# Cross-Sectional Associations between Body Size, Circulating Sex-Steroid Hormones and IGF Components among Healthy Chinese Women

**DOI:** 10.1371/journal.pone.0137686

**Published:** 2015-09-09

**Authors:** Lauren E. McCullough, Erline E. Miller, Qiong Wang, Jia-yuan Li, Li Liu, Hui Li, Jing Zhang, Jennifer S. Smith

**Affiliations:** 1 Department of Epidemiology, Gillings School of Global Public Health, University of North Carolina-Chapel Hill, 135 Dauer Drive, Chapel Hill, North Carolina, 27599, United States of America; 2 Department of Epidemiology and Biostatistics, West China School of Public Health, Sichuan University, 16 Ren Min Nan Lu, Chengdu, 610041, P.R. China; 3 The Comprehensive Guidance Center of Women's Health, Chengdu Women’s and Children’s Central Hospital, Shi Ye Street, Qingyang District, Chengdu, 610015, P.R. China; 4 Lineberger Comprehensive Cancer Center, 101 Manning Drive, Chapel Hill, North Carolina, 27514, United States of America; The Institute of Cancer Research, UNITED KINGDOM

## Abstract

The incidence of breast cancer has increased in Asian countries and rates of hormone receptor (HR) negative breast cancer exceed those of Western countries. Epidemiologic data suggest that the association between body size and BC risk may vary by HR status, and could differ geographically. While body size may influence BC risk by moderating the synthesis and metabolism of circulating sex-steroid hormones, insulin-like growth factor (IGF)-1 and related binding proteins, there is a dearth of literature among Asian women. We aimed to examine these specific associations in a sample of Chinese women. In Sichuan Province 143 women aged ≥40 years were recruited through outpatient services (2011–2012). Questionnaires, anthropometric measurements, and blood samples were utilized for data collection and linear regression was applied in data analyses. Among women <50 years we observed a non-monotonic positive association between body mass index (BMI) and 17β-estradiol, and a reversed J-shaped association between BMI and IGF-1 (*p* ≤0.05). We observed similar associations between waist-to-hip ratio and these markers. Our finding of augmented IGF-1 among women with low body mass may have implications for understanding breast tumor heterogeneity in diverse populations and should be evaluated in larger prospective studies with cancer outcomes.

## Introduction

The incidence of breast cancer has increased rapidly in Asian countries over the last several decades [1], although it remains lower than the West. The age distribution of breast cancer in Asia varies drastically from Western countries. Peak incidence occurs between ages 40 and 50 among Asian women and between ages 60 and 70 among women from Western countries [1]. Interestingly, differences in the shape of the age-incidence curve mirror differences in the age-incidence distribution for estrogen receptor (ER)+ and ER- breast cancers. Yasui and Potter showed that among Western women the linear increase in breast cancer incidence after menopause was largely driven by ER+ status and that among Asian populations the age-incidence curve closely mimicked that for ER- breast cancer, with risk diminishing after menopause [2]. Geographic differences in age distribution and ER status may be accounted for by differences in demographic, biologic, genetic, and lifestyle characteristics. For example, studies show that compared to ER+ breast tumors, ER- cancers are not strongly associated with traditional epidemiologic risk factors (i.e. parity, age at first birth, and body size) [3, 4].

Epidemiological studies have consistently shown that the association between obesity and breast cancer risk may vary by menopausal status [5], with positive associations observed for postmenopausal women [6, 7], and inverse associations observed among premenopausal women [8, 9]. Similarly, there is evidence to suggest that the obesity-breast cancer association may vary by hormone receptor status [4, 5]. A 2011 pooled study conducted among approximately 35,000, primarily Caucasian women revealed that the inverse association between obesity and premenopausal breast cancer may be restricted to women with ER+/progesterone receptor (PR)+ tumors [10]. There appeared to be a lack of protection for ER-/PR- cancers and more specifically, the triple negative phenotype (ER-, PR-, and human epidermal growth factor receptor [HER]-2). In contrast, a large multi-center study in China showed that women with a median age of 48 years who were classified as obese (BMI ≥25) were less likely to be triple negative [11], although results were not stratified by age or menopausal status. Collectively, these data suggest that the etiology for breast cancer among Chinese women differs from that of Western populations, even within intrinsic subtypes (e.g. triple negative breast cancer). Given almost a fourth of breast cancer diagnoses in China are triple negative [11], compared to approximately 15% in the West [10], uncovering the mechanisms of action for traditional breast cancer risk factors, such as body size, is of paramount importance.

It has been hypothesized that excessive body weight may influence breast cancer risk by moderating the synthesis and metabolism of circulating sex steroid hormones, insulin-like growth factor (IGF)-1 and related binding proteins (e.g. sex hormone-binding globulin [SHBG] and IGF binding protein [IGFBP]-3) [12, 13]. Obesity has been positively associated with estrogen in postmenopausal women and inversely associated with SHBG in both pre and postmenopausal women [12, 14–18]. Moreover, adiposity has been linked to elevated serum free fatty acids which may enhance IGF-1 and reduce IGFBP [13, 19]. While several studies have reported associations between indicators of body size, sex-steroid hormones and components of the IGF axis (IGF-1 and IGFBP-3), findings are inconsistent and primarily limited to White women [20–24]. Few studies have examined associations among Asian women, particularly premenopausal women, who are at greatest risk for breast cancer [25].

The purpose of this cross-sectional study was to characterize the association between body size (body mass index [BMI] and waist-to-hip ratio [WHR]) with circulating sex-steroid hormones (17β-estradiol, testosterone, and progesterone), SHBG, IGF-1 and IGFBP-3 in a sample of 143 Chinese women. Given the biologic link between sex-steroid and the IGF axis, we also describe the cross-sectional relationships between these biomarkers.

## Methods

### Ethics Statement

The study protocol was approved by the Institutional Review Board at Sichuan University. Written informed consents were obtained for all participants prior to completing the study questionnaire and donating blood samples.

### Study Population

Women aged 40 years and older were recruited through the outpatient service at the Comprehensive Guidance Center of Women's Health, Chengdu Women’s and Children’s Central Hospital between September 2011 and July 2012. This study included women of Han ethnicity, who had been living in Sichuan Province for over 20 years, and who had no history of bilateral ovariectomy, hormone replacement therapy, or perimenopausal complaints. Women with insulin-dependent diabetes mellitus or a diagnosed/history of malignancy, including breast, liver or ovarian cancer, at baseline were excluded.

### Data Collection

Of 279 eligible women, 51.3% completed both the questionnaire and donated a blood sample (N = 143). Menstruating women had blood samples collected at interview, between the 3^rd^ and 5^th^ day from onset of menstruation, to account for periodical variation in sex hormones across the menstrual cycle. Non-menstruating women provided blood samples at their scheduled interview date. For all women, samples were collected between 10:00am and 11:00am. Information on socio-demographic and reproductive characteristics was collected using a structured questionnaire, and a trained nurse obtained anthropometric data (e.g. height, weight, waist and hip) for each participant. The Questionnaire of Health Related Dietary Habits, a semi-quantitative dietary questionnaire, was used to assess participants’ long-term (≥5 years) dietary habits. The reliability and validity of the dietary questionnaire has been previously described [26].

Measurements of height (m) and weight (kg) were used to calculate BMI (weight [kg]/height [m^2^]) for each woman. Categories of <18.50 kg/m^2^, 18.50–22.99 kg/m^2^, and ≥23 kg/m^2^ were used based on World Health Organization recommendations for Asians [27]. We additionally calculated WHR (waistline [cm]/hipline [cm]) for each participant. We dichotomized WHR at the median (0.8165) to create low and high risk groups.

### Serum Biomarkers

Whole blood samples of 3 mL were withdrawn and then immediately transported to the Medical Diagnostic Laboratory, Chengdu Women’s and Children’s Central Hospital, where samples stood for 2 hours and then spun at 2500 g for 15 minutes. Serum was subsequently extracted and stored at -70°C until analysis. Assays for IGF-1, IGFBP-3, and SHBG were measured using enzyme-linked immunosorbent assay kits (Diagnostic Systems Laboratories, Webster, TX) according to manufacturer’s instructions. Circulating 17β-estradiol, testosterone, and progesterone levels were quantified by chemiluminescent immunoassay on the Immulite Analyzer (Siemens Medical Solutions Diagnostics, Los Angeles, CA).

Duplicate aliquots from each blood sample were analyzed, and the average of the two measurements was used for data analyses. The intra- and inter-assay coefficients of variation (CVs) were as follows: 4.1% and 12.8% for IGF-1 (concentration of 110 ng/ml); 4.9% and 5.4% for IGFBP-3 (concentration of 4,900 ng/ml); and 3.1% and 5.8% for SHBG (concentration of 36 ng/ml), respectively. Two blinded quality control (QC) samples were included for all biomarkers and mean intra-essay CVs for 17β-estradiol, testosterone, and progesterone were 6.0%, 4.9% and 8.1%, respectively.

### Statistical Analyses

All biomarker data were log transformed to reduce departures from the normal distribution. Additionally we describe distributions for all biomarkers using the geometric mean (95% confidence interval [CI]).

A priori, we aimed to uncover associations between markers of body size and biomarkers related to sex-steroid hormones and components of the IGF axis among premenopausal women. These analyses were therefore restricted to study participants aged <50 years (N = 104) and data among the 39 women age ≥50 years are included in the supplement. The results using self-reported menopausal status did not vary from those using age based proxies. Independent-sample t-tests [28] were used to compare markers of body size and logarithm distributions of serum biomarkers between the two age subgroups. Pairwise correlations between biomarkers and body size indices were also calculated [28]. We implemented linear regression [28] to estimate geometric mean (95% CI) biomarker levels within categories of body size, as well as to estimate the linear association between body size and relevant biomarkers.

For regression analyses, potential confounders were identified based on the analysis of causal diagrams [29]. They included total energy (categorical), passive smoking (yes/no), oral contraceptive estrogen use (yes/no), age at menarche (categorical), parity (categorical), age at first birth (categorical), duration of breastfeeding (categorical), and history of benign breast disease (yes/no). Covariates that were significantly associated with body size and sex-steroid hormones or IGF biomarkers were adjusted for in the final model. None of the covariates met our criteria. We therefore present unadjusted (crude) models. All analyses were conducted using Stata 13.0.

## Results

### Descriptive Statistics

All 143 women were of Han nationality and the average age of study participants was 46.4 years (standard deviation 4.9 years). The majority of participants (70.6%, N = 101) had finished high school, and 91.7% (N = 131) of them had an income level higher than the poverty criteria in Chengdu city (1500 RMB/month). Among women aged <50 (N = 104), mean age, BMI, and WHR were 43.99 ± 2.94, 22.2 ± 2.51, and 0.81 ± 0.06, respectively (Table 1). The mean age, BMI, and WHR among women aged ≥50 (N = 39) were 52.92 ± 2.77, 22.37 ± 2.60, and 0.83 ± 0.05, respectively (S1 Table). The majority of sex-steroid and IGF markers did not vary greatly between the two age groups, although we observed differences by age for levels of 17β-estradiol (p = 0.003). Geometric mean (95% CI) 17β-estradiol levels (pg/mL) were 57.78 (46.90, 71.17) and 30.52 (20.66, 45.08) for women aged <50 and ≥50 years, respectively.

**Table 1:** Descriptive statistics of age, anthropometric factors, sex-steroid and insulin resistance biomarkers among study participants age < 50 years.

	(n = 104)
Measurement^a^	statistical distribution
Age (years)	43.99 ± 2.94
Body Mass Index (kg/m^2^)	22.20 ± 2.51
Waist-to-hip Ratio	0.81 ± 0.06
Estradiol (pg/mL)	57.78 (46.90, 71.17)
Progesterone (ng/mL)	0.45 (0.35, 0.57)
Testosterone (ng/mL)	0.24 (0.20, 0.28)
Sex hormone binging globulin (ng/mL)	29.81 (25.32, 35.09)
Insulin-like growth factor-1 (ng/mL)	168.03 (144.31, 195.66)
Insulin-like growth factor binding protein-3 (ng/mL)	1837.54 (1642.92, 2055.22)

^a^ Age, body mass index and waist-to-hip reported as mean ± standard deviation; Biomarkers reported as geometric mean (95% confidence interval).

### Correlations between biomarkers and indicators of body size

Partial correlation coefficients between sex-steroid hormones, SHBG, IGF-1, IGFBP-3 and anthropometric variables among women age < 50 years and ≥ 50 years are shown in Table 2 and S2 Table, respectively. Overall, we observed weak positive correlations between sex-steroid hormones. Among women ≥50 years, testosterone was significantly correlated with both 17β-estradiol (*r* = 0.45, p<0.01) and progesterone (*r* = 0.36, p<0.05). With the exception of progesterone, SHBG showed weak inverse correlations with sex-steroid hormones in both age groups. We observed no strong correlations between IGF-1 and any of the sex-steroid hormones. However, we did observe strong positive correlations (*r*>0.80) between IGF-1 and SHBG in both age groups (p<0.01). IGFBP-3 also had direct correlations with IGF-1 (p<0.05) although they were lower in magnitude (<50: *r* = 0.26, ≥50: *r* = 0.33). Finally we observed a moderate inverse correlation between IGFBP-3 and 17β-estradiol among women ≥50 years (*r* = -0.43, p<0.05).

**Table 2:** Partial correlation coefficients between sex-steroid hormones, sex-hormone binding globulin, insulin-like growth factor components and anthropometric variables, among participants age < 50 years.

	E2	P	T	SHBG	IGF-1	IGFBP-3	Waist-to-hip ratio	Body mass index
Estradiol (E2)	1	-0.19	0.00	-0.10	-0.11	-0.06	0.16	0.08
Progesterone (P)	—	1	0.17	0.09	0.06	0.12	-0.06	0.02
Testosterone (T)	—	—	1	-0.01	0.04	0.1	-0.08	0.19
Sex-hormone binding globulin (SHBG)	—	—	—	1	0.83**	0.09	-0.21*	-0.05
Insulin-like growth factor-1 (IGF-1)	—	—	—	—	1	0.26*	-0.18	-0.03
Insulin-like growth binding protein-3 (IGFBP-3)	—	—	—	—	—	1	-0.18	-0.20*

*p<0.05

**p<0.01.

Among women <50 years, we observed weak inverse correlations between sex-steroid hormones and WHR. Conversely, we observed weak positive correlations between sex hormones and BMI (Table 2). Both measures of body size were inversely correlated with SHBG, IGF-1 and IGFBP-3. Significant, but weak, correlations were observed for WHR and SHBG (*r* = -0.21, p<0.05) as well as BMI and IGFBP-3 (*r* = -0.20, p<0.05). We observed no clear patterns among women ≥ 50 years (S2 Table).

### Associations between biomarkers and indicators of body size

Adjusted geometric means for sex-steroid hormones, SHBG, IGF-1 and IGFBP-3 were calculated within BMI (kg/m²) categories of <18.50, 18.50–22.99, and ≥23.00 and WHR categories of <0.8165 and ≥0.8165 (Table 3 and S3 Table). We observed a non-monotonic increase in 17β-estradiol with increasing BMI among women aged <50 (p = 0.04), while levels of SHBG and IGF-1 decreased non-monotonically with increasing BMI (p = 0.02 and 0.05, respectively). When we assessed BMI on a continuous scale we observed an inverse association with IGFBP-3 (β = -0.02, standard error (SE) = 0.01, p = 0.02). Quadratic terms were included in all models to assess departures from linearity (data not shown), but the addition of these terms did not substantially improve model fit. When we assessed these associations using WHR we found robust linear associations between WHR and both SHBG and IGF-1 (Table 3). For women aged ≥50, we observed positive association between progesterone and BMI (p = 0.002, S3 Table). Otherwise, circulating levels of 17β-estradiol, testosterone, SHBG, IGF-1, and IGFBP-3 were not associated with BMI categories in this age group. None of the markers examined appeared to be associated with WHR among women ≥50 years.

**Table 3:** Crude association between indicators of body size, sex-steroid hormones, sex-hormone binding globulin, and insulin-like growth factor components among participants age < 50 years.

	N	Estradiol (pg/mL)	Progesterone (ng/mL)	Testosterone (ng/mL)	Sex hormone binding globulin (ng/mL)	Insulin-like growth factor-1 (ng/mL)	Insulin-like growth factor binding protein-3
**Body mass index**	Geometric mean (95% confidence interval)						
<18.49 kg/m^2^	8	24.23 (11.60, 50.61)	0.23 (0.10, 0.55)	0.17 (0.09, 0.32)	63.29 (35.79, 111.92)	286.05 (166.93, 490.16)	2677.63 (1794.64, 3995.06)
18.50–22.99 kg/m^2^	61	65.74 (50.35, 85,82)	0.52 (0.38, 0.71)	0.23 (0.18, 0.29)	26.05 (21.19, 32.02)	148.38 (122.09, 180.34)	1806.19 (1562.54, 2087.82)
≥23 kg/m^2^	35	56.28 (39.58, 80.02)	0.40 (0.26, 0.60)	0.27 (0.20, 0.36)	31.75 (24.17, 41.69)	184.81 (142.85, 239.08)	1737.35 (1434.86, 2103.62)
	*p-value*	0.04	0.18	0.43	0.02	0.05	0.15
	β coefficient (standard error)						
Continuous		0.02 (0.02)	0.01 (0.02)	0.03 (0.02)	-0.01 (0.01)	-0.01 (0.01)	-0.02 (0.01)
	*p-value*	0.39	0.81	0.07	0.54	0.69	0.02
**Waist-to-hip ratio**	Geometric mean (95% confidence interval)						
<0.8165	49	48.94 (36.19, 66.20)	0.48 (0.34, 0.68)	0.23 (0.18, 0.30)	37.46 (29.74, 47.19)	193.82 (155.63, 241.39)	1852.59 (1572.52, 2182.54)
≥0.8165	55	66.98 (50.37, 89.07)	0.42 (0.30, 0.58)	0.24 (0.19, 0.31)	24.32 (19.56, 30.24)	147.96 (120.28, 182.02)	1824.24 (1562.76, 2129.46)
	*p-value*	0.14	0.57	0.84	0.01	0.08	0.89
	β coefficient (standard error)						
Continuous		1.41 (0.81)	-0.57 (0.95)	-0.55 (0.70)	-1.36 (0.63)	-1.17 (0.59)	-0.78 (0.43)
	*p-value*	0.08	0.55	0.44	0.03	0.05	0.08

## Discussion

Data from this cross-sectional analysis of Chinese women suggest a positive association between BMI and 17β-estradiol and a reversed J-shaped association between BMI and IGF-1 among women <50 years. The study also offers further evidence of an inverse association between BMI and SHBG and adds to the growing body of literature that premenopausal body size influences circulating levels of sex-steroid hormones and components of the IGF axis.

Most correlations between biomarkers were weak and non-significant. However, we observed strong positive correlations between IGF-1 and SHBG in both age groups (p<0.01), contrary to previous studies among White women [20, 30]. The reasoning behind this is not well-understood as in vitro studies have shown that hepatic SHBG synthesis may be inhibited by IGF-1 [31]. However, in our population almost half of all women used oral contraceptives, most commonly a combination of estrogen and progesterone, which are known to modulate both IGF-1 and SHBG [32, 33]. This may be one explanation accounting for the discrepancy given women with hormonal contraceptive use were excluded in most previous studies [20, 30].

Among the most commonly cited effects of obesity on circulating sex hormones are: (1) the positive association between BMI and estrogen levels in postmenopausal women; and (2) the inverse association between BMI and SHBG in both pre and postmenopausal women [12, 14–18]. Directions of association in our exploratory analysis of women ≥ 50 years are consistent with previous observations where we observed non-monotonic increases in 17β-estradiol with increasing BMI (p = 0.38), as well as increases in progesterone (p = 0.002). Body size was inversely associated with SHBG among women age <50 (p = 0.02) and, although not significant, the direction was inverse for women age ≥50. SHBG may be an important mediator of breast carcinogenesis because it binds estradiol and testosterone, both of which stimulate the proliferation of breast epithelial cells [12]. The inverse association between SHBG and body mass are likely related to enhanced insulin levels, which has been reported to inhibit hepatic synthesis of SHBG [12, 13]. Elevated estrogen levels among postmenopausal women are hypothesized to occur through the aromatization of androgens in adipose tissue [12, 34]. Compared to ovarian estrogen production, adipose mediated estrogen production is highly unregulated [12]. Unregulated estrogen production, in combination with reductions of SHBG, increases free estradiol among overweight/obese postmenopausal women [12] promoting the development of breast cancer, particularly hormone receptor-positive subtypes.

**Fig 1:**
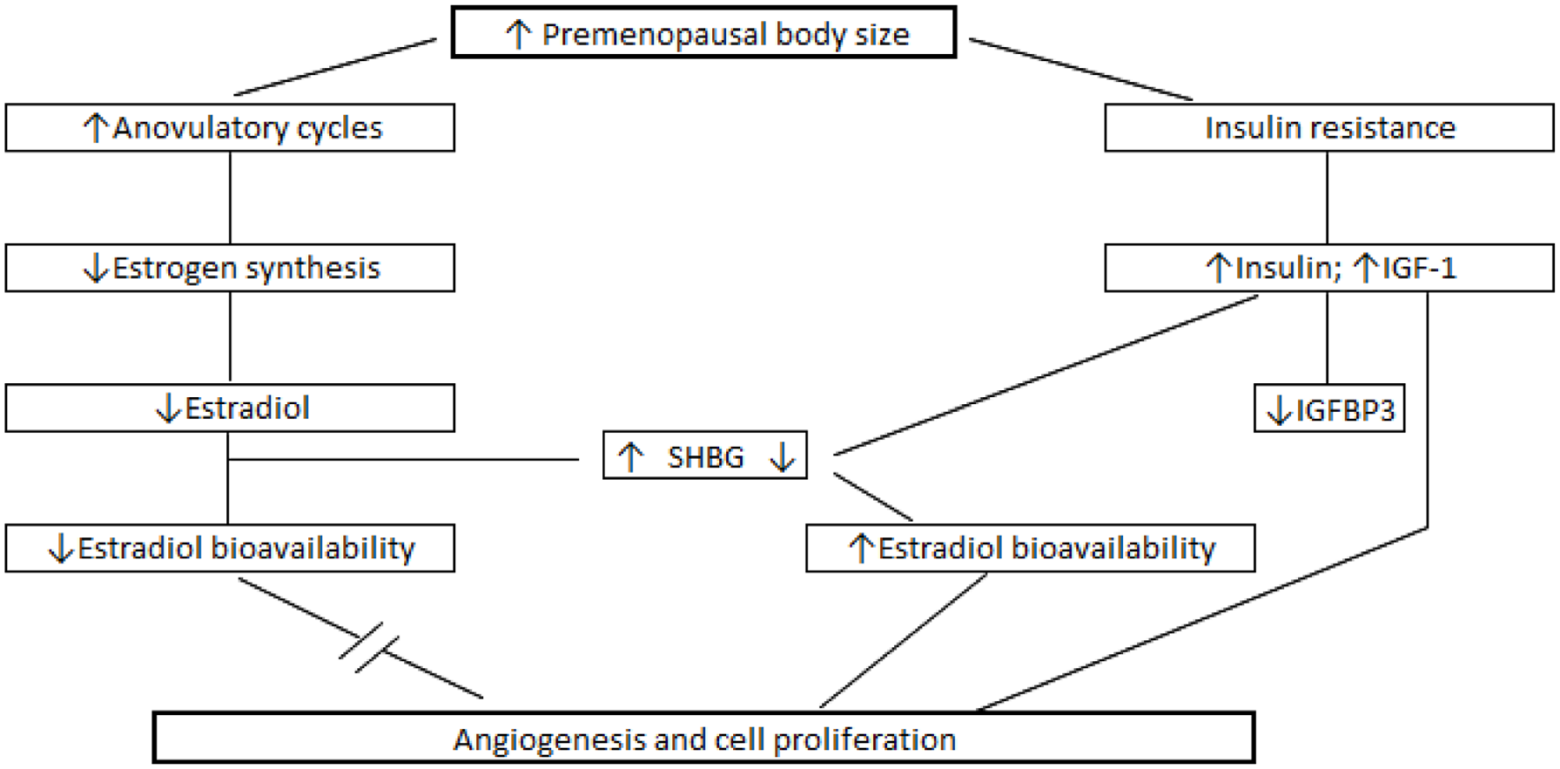
Relationship between premenopausal body size and promotion of breast cancer. *IGF-1* insulin-like growth factor-1; *SHBG* sex hormone binding globulin; *IGFBP-3* insulin-like growth factor binding protein-3.

While the exact mechanisms remain to be resolved, it is postulated that the inverse association between body size and premenopausal obesity is due to increased number of anovulatory cycles and reduced cumulative exposure to cyclic sex hormones (Fig 1) [35, 36]. This may explain the reduced risk for hormone responsive tumors among younger (age ≤ 50 years) obese women [10]. Previous studies examining the association between BMI and total estradiol levels among premenopausal women have been inconsistent. Several studies, conducted primarily among White women, reported inverse associations between premenopausal BMI and total estradiol [17, 22, 23, 37]. In contrast, two studies conducted among African-American women report positive associations between body size (WHR) and estradiol [38, 39]. We similarly observed a positive association between BMI and estradiol among women in China. In the only other study to examine the association between BMI and estradiol among premenopausal Asian women, investigators of a Japanese study observed associations similar to those reported among Whites [25]. There may be several factors accounting for this difference. The Japanese study [25] obtained samples at a single point in time without restriction to the day of the menstrual cycle, while we obtained blood between the 3^rd^ and 5^th^ day from onset of menstruation. Variation in the time at which samples were collected may have resulted in measurement error. In the Japanese study, analyses were also restricted to women with regular menstrual cycles. Controlling for a causal intermediate (anovulation) may result in spurious associations.

Although steroid hormones are known to be involved in the etiology of breast cancer, it is likely that other regulatory molecules, such as those involved in the IGF axis, work independently or in concert with estrogens to facilitate breast carcinogenesis [40]. IGF-1 has been shown to have strong mitogenic and anti-apoptotic effects on mammary cells both in vitro and in vivo; and IGFBP-3, which regulates IGF-1 bioavailability, generally exerts pro-apoptotic effects [41–43]. While the pathophysiology linking obesity to IGF-1 and IGFBP-3 levels are not completely resolved, data show that increases in visceral fat are associated with elevated levels of serum free fatty acids in the blood [44]. These fatty acids are thought to cause a reduction in glucose uptake and a rise in insulin secretion [19, 44–46] leading to a cascade of events including: (1) decreased production of IGFBPs; (2) amplified levels of IGF-1; and (3) reduced availability of SHBG (Fig 1) [13, 19]. While reduced SHBG increases the fraction of bioavailable estradiol and testosterone, increasing risk for hormone-responsive tumors; elevated IGF-1 could stimulate the growth of breast cancer cells in absence of ER or PR activation [47–49].

Previous studies have examined the association of IGF-1 and IGFBP-3 levels with various anthropometric measures; however, the results have been inconsistent [20, 21, 50–53]. Positive associations between obesity and IGFBP-3 have been reported across race and menopausal groups [24, 50, 52, 54–57]. Directions were positive among women age ≥50 years (p = 0.53), but inverse among women age <50 years (p = 0.15). With respect to IGF-1, several studies conducted among postmenopausal White women have reported an inverted U-shaped association with obesity [20, 52, 55]. Even among premenopausal White women IGF-1 is reported to be lowest for women in the lowest category of BMI [24, 55]. In contrast, one investigation showed that among premenopausal African American women IGF-1 was elevated among women with low BMI [55]. Our study results, in a Chinese population of women <50 years, are similar to the findings among premenopausal African American women. We observed a reversed J-shaped relationship between BMI and IGF-1, with the highest levels of IGF-1 observed for women with the lowest BMI (p = 0.05). To our knowledge, only one other study has reported on the association between body size and IGF-1 in a Chinese population. Among breast cancer-free control women, investigators found an inverted U-shaped association between BMI/WHR and IGF-1 [57], consistent with the hypothesis that IGF-1 increases with body weight until a threshold is reached and activation of a negative feedback loop decreases hepatic IGF-1 production [19]. However, this study did not examine the association among premenopausal controls similar to the female population in our present study.

Our study has several limitations. A relatively limited sample may have restricted our ability to ascertain precise associations between sex-steroid hormones or IGF components, and measures of body size. However, to our knowledge, this is the first study to explore these associations among young Chinese women. Our study was limited to capturing hormone levels at a single time point and may not reflect a woman’s lifetime exposure. Moreover, cyclic variations in hormones across the menstrual cycle often make studies among premenopausal women difficult. We attempted to reduce variability by obtaining blood samples between the 3^rd^ and 5^th^ day from the onset of menstruation. This study did not exclude oral contraceptive users which may lead to spurious associations. While oral estrogens have been shown to decrease IGF-1 concentrations and may confound the association between IGF-1 and body size [58], additional analyses did not indicate that our results were differential with respect to oral contraceptive use (data not shown). Finally, although we had a limited panel of biomarkers, multiple comparisons may have led to chance findings. These data should thereby be interpreted with caution.

Our finding of augmented IGF-1 among women with low body mass may have implications for understanding breast tumor heterogeneity in diverse populations and should be evaluated in larger prospective studies with cancer outcomes. It is of interest to understand how body weight, a potentially modifiable lifestyle factor, is related to IGF-1 and sex-steroid hormone concentrations given their independent associations with breast cancer risk and the increasing prevalence of obesity in developing countries [59]. Further, it is unclear if there are race/ethnic differences in the relationship between body size, sex hormones, and components of the IGF axis. Additional studies may not only improve our understanding of the mechanisms relating BMI and hormone levels to breast cancer risk, but may explain racial/geographic distributions of breast cancer subtypes.

## Supporting Information

S1 Table Descriptive statistics of age, anthropometric factors, sex-steroid and insulin resistance biomarkers among study participants age ≥ 50 years. (DOCX)

S2 Table Partial correlation coefficients between sex-steroid hormones, sex-hormone binding globulin, insulin-like growth factor components and anthropometric variables, among participants age ≥ 50. (DOCX)

S3 Table Crude association between indicators of body size, sex-steroid hormones, sex-hormone binding globulin, and insulin-like growth factor components among participants age < 50 years. (DOCX)

## References

[1] Green M, Raina V. Epidemiology, screening and diagnosis of breast cancer in the Asia–Pacific region: Current perspectives and important considerations. Asia-Pacific Journal of Clinical Oncology. 2008;4:S5–S13.

[2] Yasui Y, Potter JD. The shape of age-incidence curves of female breast cancer by hormone-receptor status. Cancer Causes Control. 1999;10(5):431–7. 10530614 10.1023/a:1008970121595

[3] Setiawan VW, Monroe KR, Wilkens LR, Kolonel LN, Pike MC, Henderson BE. Breast cancer risk factors defined by estrogen and progesterone receptor status: the multiethnic cohort study. Am J Epidemiol. 2009;169(10):1251–9. 10.1093/aje/kwp036 19318616 PMC2727208

[4] Althuis MD, Fergenbaum JH, Garcia-Closas M, Brinton LA, Madigan MP, Sherman ME. Etiology of hormone receptor-defined breast cancer: a systematic review of the literature. Cancer Epidemiol Biomarkers Prev. 2004;13(10):1558–68. 15466970

[5] Chen WY, Colditz GA. Risk factors and hormone-receptor status: epidemiology, risk-prediction models and treatment implications for breast cancer. Nat Clin Pract Oncol. 2007;4(7):415–23. 17597706 10.1038/ncponc0851

[6] Lahmann PH, Hoffmann K, Allen N, van Gils CH, Khaw KT, Tehard B, et al Body size and breast cancer risk: findings from the European Prospective Investigation into Cancer And Nutrition (EPIC). Int J Cancer. 2004;111(5):762–71. 15252848 10.1002/ijc.20315

[7] Colditz GA. Epidemiology of breast cancer. Findings from the nurses' health study. Cancer. 1993;71(4 Suppl):1480–9. 8431884 10.1002/cncr.2820710413

[8] Ursin G, Longnecker MP, Haile RW, Greenland S. A meta-analysis of body mass index and risk of premenopausal breast cancer. Epidemiology. 1995;6(2):137–41. 7742399 10.1097/00001648-199503000-00009

[9] van den Brandt PA, Spiegelman D, Yaun SS, Adami HO, Beeson L, Folsom AR, et al Pooled analysis of prospective cohort studies on height, weight, and breast cancer risk. Am J Epidemiol. 2000;152(6):514–27. 10997541 10.1093/aje/152.6.514

[10] Yang XR, Chang-Claude J, Goode EL, Couch FJ, Nevanlinna H, Milne RL, et al Associations of breast cancer risk factors with tumor subtypes: a pooled analysis from the Breast Cancer Association Consortium studies. J Natl Cancer Inst. 2011;103(3):250–63. 10.1093/jnci/djq526 21191117 PMC3107570

[11] Song Q, Huang R, Li J, Fan J, Zheng S, Zhang B, et al The diverse distribution of risk factors between breast cancer subtypes of ER, PR and HER2: a 10-year retrospective multi-center study in China. PloS one. 2013;8(8):e72175 10.1371/journal.pone.0072175 23977244 PMC3748061

[12] Key TJ, Allen NE, Verkasalo PK, Banks E. Energy balance and cancer: the role of sex hormones. Proc Nutr Soc. 2001;60(1):81–9. 11310427 10.1079/pns200068

[13] Kaaks R, Lukanova A. Energy balance and cancer: the role of insulin and insulin-like growth factor-I. Proc Nutr Soc. 2001;60(1):91–106. 11310428 10.1079/pns200070

[14] Wei S, Schmidt MD, Dwyer T, Norman RJ, Venn AJ. Obesity and menstrual irregularity: associations with SHBG, testosterone, and insulin. Obesity (Silver Spring). 2009;17(5):1070–6. 19180069 10.1038/oby.2008.641

[15] Iwasaki M, Otani T, Inoue M, Sasazuki S, Tsugane S. Body size and risk for breast cancer in relation to estrogen and progesterone receptor status in Japan. Ann Epidemiol. 2007;17(4):304–12. 17174568 10.1016/j.annepidem.2006.09.003

[16] Bernstein L. Epidemiology of endocrine-related risk factors for breast cancer. J Mammary Gland Biol Neoplasia. 2002;7(1):3–15. 12160084 10.1023/a:1015714305420

[17] Freeman EW, Sammel MD, Lin H, Gracia CR. Obesity and reproductive hormone levels in the transition to menopause. Menopause. 2010;17(4):718–26. 20216473 10.1097/gme.0b013e3181cec85d PMC2888623

[18] Suzuki R, Rylander-Rudqvist T, Ye W, Saji S, Wolk A. Body weight and postmenopausal breast cancer risk defined by estrogen and progesterone receptor status among Swedish women: A prospective cohort study. Int J Cancer. 2006;119(7):1683–9. 16646051 10.1002/ijc.22034

[19] Calle EE, Kaaks R. Overweight, obesity and cancer: epidemiological evidence and proposed mechanisms. Nat Rev Cancer. 2004;4(8):579–91. 15286738 10.1038/nrc1408

[20] Lukanova A, Lundin E, Zeleniuch-Jacquotte A, Muti P, Mure A, Rinaldi S, et al Body mass index, circulating levels of sex-steroid hormones, IGF-I and IGF-binding protein-3: a cross-sectional study in healthy women. Eur J Endocrinol. 2004;150(2):161–71. 14763914 10.1530/eje.0.1500161

[21] Lukanova A, Soderberg S, Stattin P, Palmqvist R, Lundin E, Biessy C, et al Nonlinear relationship of insulin-like growth factor (IGF)-I and IGF-I/IGF-binding protein-3 ratio with indices of adiposity and plasma insulin concentrations (Sweden). Cancer Causes Control. 2002;13(6):509–16. 12195640 10.1023/a:1016392129279

[22] Tworoger SS, Eliassen AH, Missmer SA, Baer H, Rich-Edwards J, Michels KB, et al Birthweight and body size throughout life in relation to sex hormones and prolactin concentrations in premenopausal women. Cancer Epidemiol Biomarkers Prev. 2006;15(12):2494–501. 17164375 10.1158/1055-9965.EPI-06-0671

[23] Key TJ, Appleby PN, Reeves GK, Travis RC, Alberg AJ, Barricarte A, et al Sex hormones and risk of breast cancer in premenopausal women: a collaborative reanalysis of individual participant data from seven prospective studies. Lancet Oncol. 2013;14(10):1009–19. 10.1016/S1470-2045(13)70301-2 23890780 PMC4056766

[24] Voskuil DW, Bueno de Mesquita HB, Kaaks R, van Noord PA, Rinaldi S, Riboli E, et al Determinants of circulating insulin-like growth factor (IGF)-I and IGF binding proteins 1–3 in premenopausal women: physical activity and anthropometry (Netherlands). Cancer Causes Control. 2001;12(10):951–8. 11808715 10.1023/a:1013708627664

[25] Nagata C, Wada K, Nakamura K, Hayashi M, Takeda N, Yasuda K. Associations of body size and reproductive factors with circulating levels of sex hormones and prolactin in premenopausal Japanese women. Cancer Causes Control. 2011;22(4):581–8. 10.1007/s10552-011-9731-x 21287259

[26] Wang Q, Li H, Tao P, Wang YP, Yuan P, Yang CX, et al Soy isoflavones, CYP1A1, CYP1B1, and COMT polymorphisms, and breast cancer: a case-control study in southwestern China. DNA Cell Biol. 2011;30(8):585–95. 10.1089/dna.2010.1195 21438753

[27] WHO Expert Consultation: Appropriate body-mass index for Asian populations and its implications for policy and intervention strategies. Lancet. 2004;363:157–63. 14726171 10.1016/S0140-6736(03)15268-3

[28] Selvin S. Statistical analysis of epidemiologic data. 3rd ed New York, NY: Oxford University Press; 2004.

[29] Greenland S, Brumback B. An overview of relations among causal modelling methods. Int J Epidemiol. 2002;31(5):1030–7. 12435780 10.1093/ije/31.5.1030

[30] Missmer SA, Spiegelman D, Bertone-Johnson ER, Barbieri RL, Pollak MN, Hankinson SE. Reproducibility of plasma steroid hormones, prolactin, and insulin-like growth factor levels among premenopausal women over a 2- to 3-year period. Cancer Epidemiol Biomarkers Prev. 2006;15(5):972–8. 16702379 10.1158/1055-9965.EPI-05-0848

[31] Kalme T, Koistinen H, Loukovaara M, Koistinen R, Leinonen P. Comparative studies on the regulation of insulin-like growth factor-binding protein-1 (IGFBP-1) and sex hormone-binding globulin (SHBG) production by insulin and insulin-like growth factors in human hepatoma cells. J Steroid Biochem Mol Biol. 2003;86(2):197–200. 14568572 10.1016/s0960-0760(03)00268-1

[32] Panzer C, Wise S, Fantini G, Kang D, Munarriz R, Guay A, et al Impact of oral contraceptives on sex hormone-binding globulin and androgen levels: a retrospective study in women with sexual dysfunction. J Sex Med. 2006;3(1):104–13. 16409223 10.1111/j.1743-6109.2005.00198.x

[33] Blackmore KM, Wong J, Knight JA. A cross-sectional study of different patterns of oral contraceptive use among premenopausal women and circulating IGF-1: implications for disease risk. BMC Womens Health. 2011;11:15 10.1186/1472-6874-11-15 21599947 PMC3123282

[34] Muti P. The role of endogenous hormones in the etiology and prevention of breast cancer: the epidemiological evidence. Ann N Y Acad Sci. 2004;1028:273–82. 15650252 10.1196/annals.1322.031

[35] Peacock SL, White E, Daling JR, Voigt LF, Malone KE. Relation between obesity and breast cancer in young women. Am J Epidemiol. 1999;149(4):339–46. 10025476 10.1093/oxfordjournals.aje.a009818

[36] Tehard B, Clavel-Chapelon F. Several anthropometric measurements and breast cancer risk: results of the E3N cohort study. Int J Obes (Lond). 2006;30(1):156–63. 16231021 10.1038/sj.ijo.0803133 PMC1903368

[37] Verkasalo PK, Thomas HV, Appleby PN, Davey GK, Key TJ. Circulating levels of sex hormones and their relation to risk factors for breast cancer: a cross-sectional study in 1092 pre- and postmenopausal women (United Kingdom). Cancer Causes Control. 2001;12(1):47–59. 11227925 10.1023/a:1008929714862

[38] Paxton RJ, King DW, Garcia-Prieto C, Connors SK, Hernandez M, Gor BJ, et al Associations between body size and serum estradiol and sex hormone-binding globulin levels in premenopausal African American women. J Clin Endocrinol Metab. 2013;98(3):E485–90. 10.1210/jc.2012-2782 23408572 PMC3590484

[39] Barnett JB, Woods MN, Rosner B, McCormack C, Longcope C, Houser RF Jr., et al Sex hormone levels in premenopausal African-American women with upper and lower body fat phenotypes. Nutr Cancer. 2001;41(1–2):47–56. 12094628 10.1080/01635581.2001.9680611

[40] Beckmann MW, Niederacher D, Schnurch HG, Gusterson BA, Bender HG. Multistep carcinogenesis of breast cancer and tumour heterogeneity. J Mol Med (Berl). 1997;75(6):429–39. 9231883 10.1007/s001090050128

[41] Sachdev D, Yee D. The IGF system and breast cancer. Endocr Relat Cancer. 2001;8(3):197–209. 11566611 10.1677/erc.0.0080197

[42] Gennigens C, Menetrier-Caux C, Droz JP. Insulin-Like Growth Factor (IGF) family and prostate cancer. Crit Rev Oncol Hematol. 2006;58(2):124–45. 16387509 10.1016/j.critrevonc.2005.10.003

[43] Ellis MJ, Jenkins S, Hanfelt J, Redington ME, Taylor M, Leek R, et al Insulin-like growth factors in human breast cancer. Breast Cancer Res Treat. 1998;52(1–3):175–84. 10066081 10.1023/a:1006127621512

[44] Lorincz AM, Sukumar S. Molecular links between obesity and breast cancer. Endocr Relat Cancer. 2006;13(2):279–92. 16728564 10.1677/erc.1.00729

[45] Kaaks R. Nutrition, hormones, and breast cancer: is insulin the missing link? Cancer Causes Control. 1996;7(6):605–25. 8932921 10.1007/BF00051703

[46] Harris NS, Winter WE. The chemical pathology of insulin resistance and the metabolic syndrome. MLO Med Lab Obs. 2004;36(10):20, 2–5. 15536804

[47] Renehan AG, Harvie M, Howell A. Insulin-like growth factor (IGF)-I, IGF binding protein-3, and breast cancer risk: eight years on. Endocr Relat Cancer. 2006;13(2):273–8. 16728563 10.1677/erc.1.01219

[48] Maiti B, Kundranda MN, Spiro TP, Daw HA. The association of metabolic syndrome with triple-negative breast cancer. Breast Cancer Res Treat. 2010;121(2):479–83. 10.1007/s10549-009-0591-y 19851862

[49] Pichard C, Plu-Bureau G, Neves ECM, Gompel A. Insulin resistance, obesity and breast cancer risk. Maturitas. 2008;60(1):19–30. 10.1016/j.maturitas.2008.03.002 18485631

[50] Allen NE, Appleby PN, Kaaks R, Rinaldi S, Davey GK, Key TJ. Lifestyle determinants of serum insulin-like growth-factor-I (IGF-I), C-peptide and hormone binding protein levels in British women. Cancer Causes Control. 2003;14(1):65–74. 12708727 10.1023/a:1022518321634

[51] DeLellis K, Rinaldi S, Kaaks RJ, Kolonel LN, Henderson B, Le Marchand L. Dietary and lifestyle correlates of plasma insulin-like growth factor-I (IGF-I) and IGF binding protein-3 (IGFBP-3): the multiethnic cohort. Cancer Epidemiol Biomarkers Prev. 2004;13(9):1444–51. 15342444

[52] Gram IT, Norat T, Rinaldi S, Dossus L, Lukanova A, Tehard B, et al Body mass index, waist circumference and waist-hip ratio and serum levels of IGF-I and IGFBP-3 in European women. Int J Obes (Lond). 2006;30(11):1623–31. 16552400 10.1038/sj.ijo.0803324

[53] Henderson KD, Goran MI, Kolonel LN, Henderson BE, Le Marchand L. Ethnic disparity in the relationship between obesity and plasma insulin-like growth factors: the multiethnic cohort. Cancer Epidemiol Biomarkers Prev. 2006;15(11):2298–302. 17119061 10.1158/1055-9965.EPI-06-0344

[54] Holmes MD, Pollak MN, Hankinson SE. Lifestyle correlates of plasma insulin-like growth factor I and insulin-like growth factor binding protein 3 concentrations. Cancer Epidemiol Biomarkers Prev. 2002;11(9):862–7. 12223430

[55] Fowke JH, Matthews CE, Yu H, Cai Q, Cohen S, Buchowski MS, et al Racial differences in the association between body mass index and serum IGF1, IGF2, and IGFBP3. Endocr Relat Cancer. 2010;17(1):51–60. 10.1677/ERC-09-0023 19786462 PMC2814999

[56] Diorio C, Brisson J, Berube S, Pollak M. Intact and total insulin-like growth factor-binding protein-3 (IGFBP-3) levels in relation to breast cancer risk factors: a cross-sectional study. Breast Cancer Res. 2008;10(3):R42 10.1186/bcr2093 18471292 PMC2481489

[57] Fair AM, Dai Q, Shu XO, Matthews CE, Yu H, Jin F, et al Energy balance, insulin resistance biomarkers, and breast cancer risk. Cancer Detect Prev. 2007;31(3):214–9. 17646056 10.1016/j.cdp.2007.04.003 PMC1994998

[58] Vongpatanasin W, Tuncel M, Wang Z, Arbique D, Mehrad B, Jialal I. Differential effects of oral versus transdermal estrogen replacement therapy on C-reactive protein in postmenopausal women. J Am Coll Cardiol. 2003;41(8):1358–63. 12706932 10.1016/s0735-1097(03)00156-6

[59] Ng M, Fleming T, Robinson M, Thomson B, Graetz N, Margono C, et al Global, regional, and national prevalence of overweight and obesity in children and adults during 1980–2013: a systematic analysis for the Global Burden of Disease Study 2013. Lancet. 2014;384(9945):766–81. 10.1016/S0140-6736(14)60460-8 24880830 PMC4624264

