# Supplementary material for: Cross-Sectional Associations between Body Size, Circulating Sex-Steroid Hormones and IGF Components among Healthy Chinese Women
Source: PLoS One. 2015 Sep 9;10(9):e0137686. doi: 10.1371/journal.pone.0137686 (PMC4564271; doi:10.1371/journal.pone.0137686)
Supplement: S1 Table — (DOCX) [file pone.0137686.s001.docx]

**S1 Table.** Descriptive statistics of age, anthropometric factors, sex-steroid and insulin resistance biomarkers among study participants age ≥ 50 years

|  | |
| --- | --- |
|  | (n=39) |
| Measurement^a^ | statistical distribution |
| Age (years) | 52.92 ± 2.77 |
| Body Mass Index (kg/m^2^) | 22.37 ± 2.60 |
| Waist-to-hip Ratio | 0.83 ± 0.05 |
| Estradiol (pg/mL) | 30.52 (20.66, 45.08) |
| Progesterone (ng/mL) | 0.32 (0.21, 0.49) |
| Testosterone (ng/mL) | 0.24 (0.17, 0.35) |
| Sex hormone binging globulin (ng/mL) | 37.34 (27.52, 50.66) |
| Insulin-like growth factor-1 (ng/mL) | 200.29 (150.55, 266.47) |
| Insulin-like growth factor binding protein-3 (ng/mL) | 1669.18 (1371.39, 2031.63) |
|  | |

^a^Age, body mass index and waist-to-hip reported as mean ± standard deviation; Biomarkers reported as geometric mean (95% confidence interval).
