# Supplementary material for: Cross-Sectional Associations between Body Size, Circulating Sex-Steroid Hormones and IGF Components among Healthy Chinese Women
Source: PLoS One. 2015 Sep 9;10(9):e0137686. doi: 10.1371/journal.pone.0137686 (PMC4564271; doi:10.1371/journal.pone.0137686)
Supplement: S2 Table — (DOCX) [file pone.0137686.s002.docx]

**S2 Table.**  Partial correlation coefficients between sex-steroid hormones, sex-hormone binding globulin, insulin-like growth factor components and anthropometric variables, among participants age ≥ 50 years

|  | | | | | | | | | | | | | | | | |
| --- | --- | --- | --- | --- | --- | --- | --- | --- | --- | --- | --- | --- | --- | --- | --- | --- |
|  | | | | | | E2 | | P | T | | SHBG | | IGF-1 | IGFBP-3 | Waist-to-hip ratio | Body mass index |
| Estradiol (E2) | | | | | | 1 | | 0.29 | 0.45** | | -0.23 | | -0.26 | -0.43* | -0.02 | 0.18 |
| Progesterone (P) | | | | | | -- | | 1 | 0.36* | | 0.05 | | -0.13 | -0.04 | 0.13 | 0.55** |
| Testosterone (T) | | | | | | -- | | -- | 1 | | -0.12 | | -0.14 | -0.04 | -0.05 | 0.26 |
| Sex-hormone binding globulin (SHBG) | | | | | | -- | | -- | -- | | 1 | | 0.89** | 0.20 | 0.05 | -0.25 |
| Insulin-like growth factor-1 (IGF-1) | | | | | | -- | | -- | -- | | -- | | 1 | 0.33** | 0.23 | -0.22 |
| Insulin-like growth binding protein-3 (IGFBP-3) | | | | | | -- | | -- | -- | | -- | | -- | 1 | 0.08 | 0.09 |
|  |  |  |  |  |  | |  | | |  | |  |  |  |  |  |
|  |  |  |  |  |  | |  | | |  | |  |  |  |  |  |

*p<0.05

**p<0.01
