# Supplementary material for: Cross-Sectional Associations between Body Size, Circulating Sex-Steroid Hormones and IGF Components among Healthy Chinese Women
Source: PLoS One. 2015 Sep 9;10(9):e0137686. doi: 10.1371/journal.pone.0137686 (PMC4564271; doi:10.1371/journal.pone.0137686)
Supplement: S3 Table — (DOCX) [file pone.0137686.s003.docx]

**S3 Table.** Crude association between indicators of body size, sex-steroid hormones, sex-hormone binding globulin, and insulin-like growth factor components among participants age < 50 years

|  | N | Estradiol (pg/mL) | Progesterone (ng/mL) | Testosterone (ng/mL) | Sex hormone binding globulin (ng/mL) | Insulin-like growth factor-1 (ng/mL) | Insulin-like growth factor binding protein-3 |
| --- | --- | --- | --- | --- | --- | --- | --- |
| **Body mass index** | Geometric mean (95% confidence interval) | | | | | | |
| <18.49 kg/m^2^ | 2 | 29.90 (5.32, 168.11) | 0.27 (0.05, 1.34) | 0.20 (0.04, 1.01) | 61.13 (15.75, 237.18) | 278.75 (79.58, 976.33) | 1158.37 (481.62, 2786.09) |
| 18.50-22.99 kg/m^2^ | 23 | 24.59 (14.78, 40.92) | 0.19 (0.12, 0.30) | 0.20 (0.12, 0.32) | 41.29 (27.68, 61.59) | 232.94 (160.96, 337.11) | 1803.10 (1391.96, 2335.68) |
| ≥23 kg/m^2^ | 14 | 43.64 (22.72, 83.82) | 0.81 (0 .44, 1.48) | 0.33 (0.18, 0.61) | 29.49 (17.67, 49.23) | 149.09 (92.83, 239.44) | 1549.17 (1111.84, 2158.54) |
|  | *p-value* | 0.38 | 0.002 | 0.42 | 0.44 | 0.29 | 0.53 |
|  | β coefficient (standard error) | | | | | | |
| **Continuous** |  | 0.03 (0.03) | 0.10 (0.03) | 0.05 (0.03) | -0.03 (0.03) | -0.03 (0.24) | 0.01 (0.02) |
|  | *p-value* | 0.35 | 0.002 | 0.09 | 0.23 | 0.24 | 0.55 |
| **Waist-to-hip ratio** | Geometric mean (95% confidence interval) | | | | | | |
| <0.8165 | 19 | 37.21 (21.27, 65.11) | 0.36 (0.20, 0.66) | 0.30 (0.18, 0.51) | 36.05 (23.14, 56.15) | 172.04 (114.32, 258.91) | 1593.32 (1198.50, 2118.20) |
| ≥0.8165 | 20 | 25.28 (14.65, 43.61) | 0.29 (0.16, 0.52) | 0.19 (0.12, 0.32) | 38.60 (25.06, 59.46) | 231.42 (155.37, 344.69) | 1744.58 (1321.78, 2302.64) |
|  | *p-value* | 0.32 | 0.61 | 0.21 | 0.82 | 0.30 | 0.65 |
|  | β coefficient (standard error) | | | | | | |
| **Continuous** |  | -0.17 (1.81) | 1.32 (1.94) | -0.30 (1.70) | 0.51 (1.42) | 1.79 (1.29) | 0.42 (0.91) |
|  | *p-value* | 0.92 | 0.50 | 0.86 | 0.72 | 0.17 | 0.65 |
